# Combined evaluation of sexually transmitted infections in HIV-infected pregnant women and infant HIV transmission

**DOI:** 10.1371/journal.pone.0189851

**Published:** 2018-01-05

**Authors:** Kristina Adachi, Jiahong Xu, Nava Yeganeh, Margaret Camarca, Mariza G. Morgado, D. Heather Watts, Lynne M. Mofenson, Valdilea G. Veloso, Jose Henrique Pilotto, Esau Joao, Glenda Gray, Gerhard Theron, Breno Santos, Rosana Fonseca, Regis Kreitchmann, Jorge Pinto, Marisa M. Mussi-Pinhata, Mariana Ceriotto, Daisy Maria Machado, Yvonne J. Bryson, Beatriz Grinsztejn, Jack Moye, Jeffrey D. Klausner, Claire C. Bristow, Ruth Dickover, Mark Mirochnick, Karin Nielsen-Saines, for the NICHD HPTN 040 Study Team

**Affiliations:** 1 David Geffen UCLA School of Medicine, Los Angeles, CA, United States of America; 2 Westat, Rockville, MD, United States of America; 3 Fundacao Oswaldo Cruz (FIOCRUZ), Rio de Janeiro, RJ, Brazil; 4 Office of the Global AIDS Coordinator, U.S. Department of State, Washington D.C., United States of America; 5 Elizabeth Glaser Pediatric AIDS Foundation, Washington D.C., United States of America; 6 Hospital Geral de Nova Iguaçu, Nova Iguaçu, RJ, Brazil; 7 Hospital Federal dos Servidores do Estado, Rio de Janeiro, RJ, Brazil; 8 SAMRC and Perinatal HIV Research Unit, University of Witwatersrand, Johannesburg, South Africa; 9 Stellenbosch University/Tygerberg Hospital, Cape Town, South Africa; 10 Hospital Conceicao, Porto Alegre, RS, Brazil; 11 Hospital Femina, Porto Alegre, RS, Brazil; 12 Irmandade da Santa Casa de Misericordia de Porto Alegre, Porto Alegre, RS, Brazil; 13 Federal University of Minas Gerais, Belo Horizonte, MG, Brazil; 14 Ribeirão Preto Medical School, University of São Paulo, São Paulo, SP, Brazil; 15 Foundation for Maternal and Infant Health (FUNDASAMIN), Buenos Aires, Argentina; 16 Escola Paulista de Medicina-Universidade Federal de São Paulo, São Paulo, SP, Brazil; 17 Eunice Kennedy Shriver National Institute of Child Health and Human Development, National Institutes of Health, Bethesda, MD, United States of America; 18 UCLA Fielding School of Public Health, Los Angeles, CA, United States of America; 19 UCSD School of Medicine, La Jolla, CA, United States of America; 20 UC Davis School of Medicine, Davis, CA, United States of America; 21 Boston University School of Medicine, Boston, MA, United States of America; TNO, NETHERLANDS

## Abstract

**Background:**

Sexually transmitted infections (STIs) including *Chlamydia trachomatis* (CT), *Neisseria gonorrhoeae* (NG), *Treponema pallidum* (TP), and cytomegalovirus (CMV) may lead to adverse pregnancy and infant outcomes. The role of combined maternal STIs in HIV mother-to-child transmission (MTCT) was evaluated in mother-infant pairs from NICHD HPTN 040.

**Methodology:**

Urine samples from HIV-infected pregnant women during labor were tested by polymerase chain reaction (PCR) for CT, NG, and CMV. Infant HIV infection was determined by serial HIV DNA PCR testing. Maternal syphilis was tested by VDRL and confirmatory treponemal antibodies.

**Results:**

A total of 899 mother-infant pairs were evaluated. Over 30% had at least one of the following infections (TP, CT, NG, and/or CMV) detected at the time of delivery. High rates of TP (8.7%), CT (17.8%), NG (4%), and CMV (6.3%) were observed. HIV MTCT was 9.1% (n = 82 infants). HIV MTCT was 12.5%, 10.3%, 11.1%, and 26.3% among infants born to women with CT, TP, NG or CMV respectively. Forty-two percent of HIV-infected infants were born to women with at least one of these 4 infections. Women with these infections were nearly twice as likely to have an HIV-infected infant (aOR 1.9, 95% CI 1.1–3.0), particularly those with 2 STIs (aOR 3.4, 95% CI 1.5–7.7). Individually, maternal CMV (aOR 4.4 1.5–13.0) and infant congenital CMV (OR 4.1, 95% CI 2.2–7.8) but not other STIs (TP, CT, or NG) were associated with an increased risk of HIV MTCT.

**Conclusion:**

HIV-infected pregnant women identified during labor are at high risk for STIs. Co-infection with STIs including CMV nearly doubles HIV MTCT risk. CMV infection appears to confer the largest risk of HIV MTCT.

**Trial registration:**

NCT00099359.

## Introduction

*Chlamydia trachomatis* (CT), *Neisseria gonorrhoeae* (NG), and *Treponema pallidum* (TP) contribute to formidable global burden of treatable bacterial sexually transmitted infections (STIs) with nearly a quarter of a billion of new cases reported annually.[1] These treatable STIs disproportionately impact the health of pregnant women, particularly adolescents and young women in low and middle-income countries.[1–3] Untreated CT, NG, and TP are associated with adverse pregnancy outcomes including spontaneous abortion, stillbirth, preterm labor and delivery. Maternal infection with these conditions has also been associated with neonatal infections such as conjunctivitis (CT, NG), pneumonia (CT), and disseminated infection (TP, NG); TP may be particularly devastating given its associations with multi-organ involvement, failure to thrive, and neonatal death.[4–11] Apart from these bacterial STIs, other sexually transmitted infections including viruses such as cytomegalovirus (CMV)[12, 13] are often overlooked but can also have profound infant sequelae including neurodevelopmental anomalies and sensorineural hearing loss when acquired in-utero.[14, 15] CMV, which can be transmitted by close or sexual contact, is the major infectious etiology of sensorineural hearing loss and developmental delay, and congenital infection globally may be as high as 1–5%.[16–18] Routine antenatal screening for these conditions is not routine practice in many regions of the world, particularly in low and middle-income countries. These practices may have particular importance for high-risk groups such as HIV-infected pregnant women and their infants.

Given earlier findings of our HPTN 040 sub-studies evaluating the individual effects of STIs on HIV MTCT (CT and NG and HIV MTCT; TP and HIV MTCT; and CMV and HIV MTCT),[19–22] the following analysis provides a more comprehensive evaluation of the risk factors associated with these STIs (CT, NG, TP, and CMV) and the impact of these combined untreated infections on HIV MTCT in a high-risk cohort of late-presenting HIV-infected pregnant women who did not receive antiretroviral drugs (ARV) during pregnancy.

## Methods

### Study design

This study was a retrospective, cross-sectional clinical trial sub-study of the National Institute of Child Health and Human Development (NICHD) HIV Prevention Trials Network (HPTN) 040 trial, which is also known as the International Maternal Pediatric Adolescent AIDS Clinical Trials Network (IMPAACT) P1043. Clinical data and specimens were collected from NICHD/HPTN 040 (P1043), a phase 3, triple-arm, randomized, open-label, multi-center study aimed at the prevention of *intrapartum* HIV transmission to infants born to HIV-infected pregnant women, who had not received antiretroviral drugs until labor and delivery due to late diagnosis of infection.[23] Enrollment in the HPTN 040 parent study occurred from April 2004 through July 2010 with the last patients followed until 2011, which included 1684 HIV-infected pregnant women, including the majority who were diagnosed as HIV-infected at the time of labor and delivery. All mothers provided written informed consent. Enrollment occurred at multiple sites in Brazil, South Africa, Argentina, and the United States. Infants <32 weeks of age were excluded from the study. All HIV-exposed infants enrolled in the study were formula-fed. Infants were randomized at birth to one of three neonatal prophylactic ARV regimens. The primary endpoint was HIV infection status at 3 months of age.

Women were enrolled during labor and delivery with information obtained on maternal sociodemographics, obstetric history including prior prenatal care, prior stillbirths, and risk behaviors during pregnancy. Maternal plasma HIV RNA levels and T lymphocyte subsets were collected. Syphilis testing was performed using Venereal Disease Research Laboratory (VDRL) titers with confirmatory treponemal syphilis antibody tests performed, as per standard of care.[20] Infants were followed until 6 months of age for safety and toxicity monitoring in the parent study with serious adverse events (SAEs) recorded as previously described.[23]

#### HIV diagnosis

HIV testing of infants occurred within 48 hours of birth, 14 days, 4–6 weeks, 3, and 6 months of age. Confirmatory HIV DNA testing was done for positive results. Testing procedures have been previously described.[23]

#### Chlamydia, gonorrhea, and CMV testing

Stored maternal urine samples collected at the time of labor and delivery or within 48 hours of giving birth were frozen and stored at study sites. Aliquots (7 mL each) of stored frozen urine were shipped for testing at Cepheid, Sunnyvale, CA. Urines were tested for the presence of CT and NG using the Xpert® CT/NG assay. Results were reported as positive, negative or indeterminate. Indeterminate test results were repeated up to two times, and those that remained indeterminate were excluded from data analysis. Remaining 1mL aliquots of maternal and infant urines (also collected within 48 hours of delivery) were tested by qualitative Real-time PCR for CMV DNA (FOCUS Diagnostics CMV Analyte Specific Reagent), and those with positive results were tested by quantitative CMV PCR. In this study, given the limited number of maternal urines available for CMV PCR testing, primarily infant CMV urine results were used in the analysis, (although analysis was also done with maternal urine CMV PCR results when available as indicated in our Tables). The STIs were not treated in pregnancy because the women had not presented for care, and STI testing was done retrospectively on stored specimens.

#### Statistical analysis

All computations were done using SAS software v9.3 (Cary, NC, USA). Two-sample t-test or Kruskal-Wallis test was used to compare mean or median differences for continuous variables as appropriate. Chi-squared or Fisher’s exact test was used to compare differences of proportions for categorical variables. Univariate and multivariate logistic regression modeling was used to examine the relationship of HIV MTCT and maternal STI infection (CT, NG, TP, CMV) with potential risk factors respectively. The risk factors include maternal demographic, alcohol, tobacco, illegal substance use, pregnancy and delivery characteristics, and HIV-related clinical characteristics. Covariates with a p-value of 0.15 or less from univariate models were selected for the initial multivariable model. Covariates with an overall p-value <0.05 were retained in the final model.

#### Human subjects

The study was approved by the institutional review boards and national ethics committees at each of the participating study sites. The study was approved by the OHRP, the HPTN ethics board and the NICHD. It was also approved by the IRBs of the following institutions locally: Fiocruz, Rio de Janeiro, Hospital dos Servidores do Estado, Rio de Janeiro, Hospital Geral de Nova Iguacu, Rio de Janeiro, Grupo Hospitalar Conceicao in Porto Alegre (Hospital Conceicao and Hospital Femina), Santa Casa da Misericordia, Porto Alegre, Universidade Federal de Minas Gerais, Belo Horizonte, Universidade Federal de Sao Paulo, Sao Paulo and Ribeirao Preto, all in Brazil; Foundation for Maternal and Infant Health, Buenos Aires, Argentina, SAMRC and Perinatal HIV Research Unit, Soweto, South Africa, Tygerberg Hospital, Cape Town, South Africa, Boston University School of Medicine, MA, USA. UCLA IRB #02-04-86-12 approved the NICHD HPTN 040 study and was the lead IRB for the overall study. This particular study was a secondary endpoint analysis of the parent study and evaluated previously collected data and specimens from NICHD HPTN 040. As such, it received the UCLA IRB approval exemption number 14–001348.

## Results

### Baseline characteristics of mother-infant pairs

Among 1684 mother-infant pairs enrolled in the parent study, 899 pairs (53.4%) had specimens available for maternal CT, NG, TP, and infant CMV testing. Specimen availability for testing of all four potential co-infections determined inclusion in the present analysis. As such, 785 mother-infant pairs (approximately 47% of the original HPTN 040 cohort) were excluded. We evaluated potential differences in both populations and determined that both groups were very similar in most parameters (S1 Table). Most women in this analysis (86.2%) were from the Americas (Brazil, Argentina, and the U.S.) compared to South Africa (13.8%). (Table 1) The mean maternal age was 26.5 (SD 6.3) years, and the majority received some prenatal care during pregnancy (69.4%). High rates of alcohol (36.5%), tobacco (37%), and illegal substance (9.8%) usage were noted during pregnancy. There were also high rates of prior poor pregnancy outcomes reported such as stillbirth (4.8%). Median log_10_ HIV plasma viral load at the time of delivery was 4.2 (interquartile range = IQR 1.7–6.5) copies/mL, with 58% having HIV viral load >10,000 copies/mL; the median CD4 count was 465 (IQR 12–2160) cells/mm^3^. Preterm delivery (<37 weeks) occurred in 9.2% of infants, and 15.8% were of low birth weight (<2500 grams). Approximately 38.9% of infants experienced a serious adverse event (SAE) during the study period.

**Table 1:** Demographic, Baseline, and other characteristics of NICHD HPTN 040 mother-infant pairs.

	Total (N = 899)	Mean (std. dev.)	Median (min-max)
	n (col %)		
**Maternal CMV (CMV viruria)**			
Detected	23 (2.6)		
Missing	647 (72.0)		
Not detected	229 (25.5)		
**Parent HPTN 040 Study arm**			
ZDV	304 (33.8)		
ZDV+NVP	307 (34.1)		
ZDV+3TC+NFV	288 (32.0)		
**Maternal age (years)**		26.5 (6.3)	26 (14–47)
13–24	376 (41.8)		
25–29	252 (28.0)		
30 and older	271 (30.1)		
**Maternal HIV viral load, categorical (copies/mL)**			
≤400	52 (5.8)		
401 to ≤ 10,000	322 (35.8)		
10,001 to 100,000	418 (46.5)		
>100,000	104 (11.6)		
**Log**_**10**_ **maternal HIV viral load**		4.1 (0.8)	4.2 (1.7–6.5)
**Maternal CD4+ count (cells/mm**^**3**^**)**		519.3 (308.8)	465 (12–2160)
**Region**			
Americas	775 (86.2)		
South Africa	124 (13.8)		
**Mode of delivery**			
Cesarean before rupture	246 (27.4)		
Vaginal + CS after rupture	653 (72.6)		
**Maternal syphilis (TP)**			
No	821 (91.3)		
Yes	78 (8.7)		
**Maternal chlamydia (CT) or gonorrhea (NG)**			
No	726 (80.8)		
Yes	173 (19.2)		
**Maternal Chlamydia (CT)**			
No	739 (82.2)		
Yes	160 (17.8)		
**Maternal Gonorrhea (NG)**			
No	863 (96.0)		
Yes	36 (4.0)		
**CMV (Infant Congenital CMV; cCMV)**			
No	842 (93.7)		
Yes	57 (6.3)		
**Any STI (maternal TP, CT, NG, or** **cCMV positive)**			
No	626 (69.6)		
Yes	273 (30.4)		
**Prenatal care**			
No	273 (30.4)		
Yes	624 (69.4)		
**Time (hours) of membrane rupture to delivery**			
Unknown	93 (10.3)		
>24	25 (2.8)		
12–24	42 (4.7)		
6-<12	69 (7.7)		
.5-<6	192 (21.4)		
0.5	478 (53.2)		
**Alcohol use during pregnancy**			
≥1/week	139 (15.6)		
>1/month, <1/week	64 (7.2)		
≤1/month	123 (13.8)		
Never	566 (63.5)		
**Illegal substance use during pregnancy**			
Yes	88 (9.8)		
No	807 (90.2)		
**Infant death**			
No	879 (97.8)		
Yes	20 (2.2)		
**Tobacco use during pregnancy**			
>10/day	124 (13.9)		
6-10/day	63 (7.0)		
≤5/day	144 (16.1)		
Never	563 (63.0)		
**Gestation age (weeks)**		38.6 (1.6)	39 (32–42)
36 or less (preterm)	83 (9.2)		
37 or more	816 (90.8)		
**Low birth weight (grams)**		3009.4 (516.7)	3010 (1595–4410)
≥ 2500	757 (84.2)		
< 2500	142 (15.8)		
**Prior Stillbirth**			
No	855 (95.2)		
Yes	43 (4.8)		
**Any Infant Serious Adverse Event**			
No	549 (61.1)		
Yes	350 (38.9)		

Abbreviations: 3TC = lamivudine; CI, confidence interval; CMV, cytomegalovirus; cCMV = congenital CMV infection; CS = Cesarean section; CT = *Chlamydia trachomatis*; HIV, human immunodeficiency virus; NFV = nelfinavir; NG = *Neisseria gonorrhoeae*; NVP = nevirapine; OR, odds ratio; SD, standard deviation; STI = sexually transmitted infection; ZDV = zidovudine.

Rates of co-infections were high in this sub-cohort; over 30% of women had at least one of the infections of interest (TP, CT, NG, and/or CMV). High rates of TP (8.7%), CT (17.8%), and NG (4%) were found. Based on a positive infant urine CMV PCR at birth, which revealed the presence of congenital CMV (cCMV) infection and was used as a surrogate for active maternal CMV infection, 6.3% of women had CMV infection during pregnancy. (Table 1)

### Characteristics of STI positive and STI negative HIV-infected pregnant women

Characteristics of mother-infant pairs were compared for women with and without STIs (TP, CT, NG, and/or CMV). (Table 2) Most women with STIs were under 30 years of age (77.3%). Rates of STIs (35.6%) were highest among young women (13–24 years). The results from the adjusted multivariable model indicated that younger women were 1.6 to nearly two times more likely to have an STI (aOR 1.9, 95% CI 1.3–2.8 for those 13–24 years of age and aOR 1.6, 95% CI 1.1–2.5 for those 25–29 years of age) when compared with older women ages 30 years and above. Infants with any SAEs were 2.4-times more likely to born to mothers with STIs (aOR 2.4, 95% CI: 1.8–3.2). Cesearean delivery prior to rupture of membranes was less frequent in women with maternal STIs (aOR 0.55, 95% CI 0.37–0.83). (Table 2) In addition, frequent alcohol use (≥ 1/week) (OR 1.7, 95% CI 1.2–2.5), frequent tobacco use (>10/day) (OR 1.5, 95% CI 1.0–2.3), and illegal substance use (OR 1.7, 95% CI 1.1–2.6) as well as low birth weight infants (OR 1.5, 95% CI 1.0–2.2) were also associated with maternal STI-infection. However, these risk factors were not significant in the adjusted multivariate models.

**Table 2:** Correlates and outcomes associated with having Any Maternal STI [Syphilis (TP), chlamydia (CT), gonorrhea (NG) or CMV].

	STI Positive (N = 273)	STI Negative (N = 626)	Unadjusted		Adjusted	
	n (row %)	n (row %)	OR (95% CI)	p-value	OR (95% CI)	p-value
**Parent HPTN 040 Study arm**						
ZDV	91 (29.9)	213 (70.1)	1.00			
ZDV+NVP	103 (33.6)	204 (66.4)	1.18 (0.84–1.66)	0.34		
ZDV+3TC+NFV	79 (27.4)	209 (72.6)	0.88 (0.62–1.26)	0.50		
**Maternal age (years)**						
**13–24**	**134 (35.6)**	**242 (64.4)**	**1.87 (1.31–2.66)**	**0.001**	**1.93 (1.34–2.78)**	**0.0004**
**25–29**	**77 (30.6)**	**175 (69.4)**	**1.48 (1.00–2.19)**	**0.05**	**1.63 (1.09–2.45)**	**0.02**
30 and older	62 (22.9)	209 (77.1)	1.00		1.00	
**Region**						
Americas	237 (30.6)	538 (69.4)	1.00			
South Africa	36 (29.0)	88 (71.0)	0.93 (0.61–1.41)	0.73		
**Maternal HIV viral load,** **categorical (copies/mL)**						
≤400	13 (25.0)	39 (75.0)	1.00			
401 to ≤ 10,000	91 (28.3)	231 (71.7)	1.18 (0.60–2.32)	0.63		
10,001 to 100,000	131 (31.3)	287 (68.7)	1.37 (0.71–2.65)	0.35		
>100,000	36 (34.6)	68 (65.4)	1.59 (0.75–3.35)	0.22		
**Log10 of maternal viral load**	271 (30.2)	625 (69.8)	1.14 (0.96–1.36)	0.12		
**Maternal CD4+ count** **(cells/mm**^**3**^**)/100**	268 (30.3)	617 (69.7)	1.03 (0.98–1.08)	0.20		
**Mode of delivery**						
**Cesarean before rupture**	**59 (24.0)**	**187 (76.0)**	**0.65 (0.46–0.91)**	**0.01**	**0.55 (0.37–0.83)**	**0.004**
Vaginal + CS After rupture	214 (32.8)	439 (67.2)	1.00		1.00	
**Prenatal Care**						
No	95 (34.8)	178 (65.2)	1.00			
Yes	178 (28.5)	446 (71.5)	0.75 (0.55–1.01)	0.06		
**Alcohol use during pregnancy**						
**≥1/week**	**55 (39.6)**	**84 (60.4)**	**1.71 (1.16–2.51)**	**0.01**		
>1/month, <1/week	22 (34.4)	42 (65.6)	1.36 (0.79–2.36)	0.27		
≤1/month	37 (30.1)	86 (69.9)	1.12 (0.73–1.72)	0.60		
Never	157 (27.7)	409 (72.3)	1.00			
**Tobacco use during pregnancy**						
**>10/day**	**45 (36.3)**	**79 (63.7)**	**1.53 (1.01–2.30)**	**0.04**		
6-10/day	20 (31.7)	43 (68.3)	1.25 (0.71–2.19)	0.44		
**≤5/day**	**53 (36.8)**	**91 (63.2)**	**1.56 (1.06–2.30)**	**0.02**		
Never	153 (27.2)	410 (72.8)	1.00			
**Illegal substance use** **during pregnancy**						
**Yes**	**36 (40.9)**	**52 (59.1)**	**1.68 (1.07–2.63)**	**0.03**		
No	236 (29.2)	571 (70.8)	1.00			
**Infant death**						
No	268 (30.5)	611 (69.5)	1.00			
Yes	5 (25.0)	15 (75.0)	0.76 (0.27–2.11)	0.60		
**Gestation age (weeks)**						
36 or less	30 (36.1)	53 (63.9)	1.33 (0.83–2.14)	0.23		
37 or more	243 (29.8)	573 (70.2)	1.00			
**Low birth weight (grams)**						
≥ 2500	219 (28.9)	538 (71.1)	1.00			
**< 2500**	**54 (38.0)**	**88 (62.0)**	**1.51 (1.04–2.19)**	**0.03**		
**History of prior stillbirth**						
No	261 (30.5)	594 (69.5)	1.00			
Yes	12 (27.9)	31 (72.1)	0.88 (0.45–1.74)	0.7158		
**Any Infant Serious Adverse Event**						
No	128 (23.3)	421 (76.7)	1.00		1.00	
**Yes**	**145 (41.4)**	**205 (58.6)**	**2.33 (1.74–3.11)**	**< .0001**	**2.38 (1.76–3.21)**	**<0.0001**

Abbreviations: 3TC = lamivudine; CI, confidence interval; CMV, cytomegalovirus; CS = Cesarean section; CT = *Chlamydia trachomatis*; HIV, human immunodeficiency virus; NFV = nelfinavir; NG = *Neisseria gonorrhoeae*

NVP = nevirapine; OR, odds ratio; SD, standard deviation; STI = sexually transmitted infection; ZDV = zidovudine.

### Syphilis, chlamydia, gonorrhea, CMV and HIV mother-to-child transmission (MTCT)

82 infants of 899 (9.1%) HIV-infected mothers acquired HIV (*in utero* or *intrapartum*). Fifty (61%) infants were HIV-infected *in utero*, while 32 (39%) were infected *intrapartum*. Thirty-five (42.7%) HIV-infected infants were born to women with at least one of the STIs of interest (TP, CT, NG and/or CMV), whereas maternal STI rates among HIV-uninfected infants were 29.1%. (Table 3) Of the 35 HIV-infected infants born to women with STIs, 11 (31.4%) were born to women with multiple STIs, among whom *in utero* infection was the more common means of infant HIV acquisition (73%). (S2 Table includes additional details of HIV *in utero*, *intrapartum* transmission and specific maternal STIs). While HIV MTCT rates were higher among women with individual TP, CT, and NG infections compared to women without these STIs at delivery, this was not statistically significant: TP (10.3% vs 9%, p = 0.7), CT (12.5% vs 8.4%, p = 0.1), NG (11.1% vs 9%, p = 0.7). In contrast, HIV MTCT rates were significantly different in the presence of infant cCMV infection (i.e. congenital CMV infection), (26.3% vs 8%, p<0.0001)] and maternal CMV infection [(i.e. maternal CMV viruria), (30.4% vs 8.3%, p = 0.01)] compared to those without CMV. (Table 3) Infants born to women with one of these STIs (CT, NG, TP, and/or CMV) were nearly twice as likely to be HIV-infected (OR 1.8, 95% CI 1.1–2.9) compared to those born to women without these infections; these findings (aOR 1.9, 95% CI 1.1–3.0) persisted after adjusting for risk factors including maternal HIV viral load, maternal HIV log_10_ viral load, maternal CD4 count, prolonged rupture of membranes (either 12–24 hours or >24 hours), illegal substance use during pregnancy, and infant death.

**Table 3:** Relationship of Infant HIV transmission with risk factors.

	Infant HIV Positive (N = 82)	Infant HIV Negative (N = 817)	Unadjusted		Adjusted	
	n (row %)	n (row %)	OR (95% CI)	p-value	OR (95% CI)	p-value
**Maternal CMV** **(CMV viruria)**						
**Detected**	**7 (30.4)**	16 (69.6)	**4.84 (1.77–13.21)**	**0.002**	**4.44 (1.51–13.0)**	**0.01**
Missing	56 (8.7)	591 (91.3)	1.05 (0.61–1.80)	0.87	0.96 (0.54–1.70)	0.89
Not detected	**19 (8.3)**	210 (91.7)	1.00		1.00	
**Parent HPTN 040 Study arm**						
ZDV	37 (12.2)	267 (87.8)	1.00		1.00	
ZDV+NVP	22 (7.2)	285 (92.8)	0.56 (0.32–0.97)	0.04	0.52 (0.29–0.94)	0.03
ZDV+3TC+NFV	23 (8.0)	265 (92.0)	0.63 (0.36–1.08)	0.09	0.61 (0.34–1.09)	0.09
**Maternal age (years)**						
13–24	36 (9.6)	340 (90.4)	0.96 (0.57–1.62)	0.87		
25–29	19 (7.5)	233 (92.5)	0.74 (0.40–1.36)	0.33		
30 and older	27 (10.0)	244 (90.0)	1.00			
**Maternal HIV viral load,** **Categorical (copies/mL)**						
≤400	**2 (3.8)**	50 (96.2)	1.00			
401 to ≤ 10,000	19 (5.9)	303 (94.1)	1.57 (0.35–6.94)	0.55		
10,001 to 100,000	41 (9.8)	377 (90.2)	2.72 (0.64–11.59)	0.18		
**>100,000**	**20 (19.2)**	84 (80.8)	**5.95 (1.33–26.55)**	**0.02**		
**Region**						
Americas	73 (9.4)	702 (90.6)	1.00			
South Africa	9 (7.3)	115 (92.7)	0.75 (0.37–1.55)	0.44		
**Mode of delivery**						
Cesarean before rupture	19 (7.7)	227 (92.3)	0.78 (0.46–1.34)	0.37		
Vaginal + CS after rupture	63 (9.6)	590 (90.4)	1.00			
**Maternal syphilis (TP)**						
No	74 (9.0)	747 (91.0)	1.00			
Yes	8 (10.3)	70 (89.7)	1.15 (0.53–2.49)	0.72		
**Maternal chlamydia (CT) or gonorrhea (NG)**						
No	62 (8.5)	664 (91.5)	1.00			
Yes	20 (11.6)	153 (88.4)	1.40 (0.82–2.39)	0.22		
**Maternal chlamydia (CT)**						
No	62 (8.4)	677 (91.6)	1.00			
Yes	20 (12.5)	140 (87.5)	1.56 (0.91–2.67)	0.10		
**Maternal gonorrhea (NG)**						
No	78 (9.0)	785 (91.0)	1.00			
Yes	4 (11.1)	32 (88.9)	1.26 (0.43–3.65)	0.67		
**CMV (Infant Congenital CMV; cCMV)**						
No	**67 (8.0)**	775 (92.0)	1.00			
**Yes**	**15 (26.3)**	42 (73.7)	**4.13 (2.18–7.84)**	**< .0001**		
**Any STI (maternal TP, CT, NG or cCMV positive)**						
No	**47 (7.5)**	579 (92.5)	1.00		1.00	
**Yes**	**35 (12.8)**	**238 (87.2)**	**1.81 (1.14–2.88)**	**0.01**	**1.85 (1.13–3.02)**	**0.01**
**Subjects with 0, 1, 2, 3, 4** **STIs**						
0 STI	**47 (7.5)**	579 (92.5)	1.00			
1 STI	24 (10.8)	199 (89.2)	1.49 (0.89–2.49)	0.13	1.57 (0.91–2.71)	0.11
**2 STIs**	**10 (23.8)**	**32 (76.2)**	**3.85 (1.78–8.31)**	**0.001**	**3.44 (1.53–7.74)**	**0.003**
3 STIs	1 (12.5)	7 (87.5)	1.76 (0.21–14.61)	0.60	1.12 (0.13–10.1)	0.92
**Prenatal Care**						
No	22 (8.1)	251 (91.9)	1.00			
Yes	60 (9.6)	564 (90.4)	1.21 (0.73–2.02)	0.46		
**Time (hours) of membrane** **Rupture prior to Delivery**						
Unknown	7 (7.5)	86 (92.5)	1.00 (0.43–2.32)	1.00		
**>24**	**5 (20.0)**	20 (80.0)	**3.07 (1.09–8.66)**	**0.03**		
**12–24**	**8 (19.0)**	34 (81.0)	**2.89 (1.25–6.71)**	**0.01**		
6-<12	6 (8.7)	63 (91.3)	1.17 (0.47–2.89)	0.73		
.5-<6	20 (10.4)	172 (89.6)	1.43 (0.80–2.54)	0.22		
0.5	**36 (7.5)**	442 (92.5)	1.00			
**Alcohol use during pregnancy**						
> = 1/week	17 (12.2)	122 (87.8)	1.38 (0.77–2.47)	0.28		
>1/month, <1/week	5 (7.8)	59 (92.2)	0.84 (0.32–2.18)	0.72		
≤1/month	7 (5.7)	116 (94.3)	0.60 (0.26–1.35)	0.21		
Never	52 (9.2)	514 (90.8)	1.00			
**Illegal substance use** **during pregnancy**						
**Yes**	**13 (14.8)**	75 (85.2)	**1.85 (0.98–3.51)**	**0.06**	**2.32 (1.16–4.64)**	**0.02**
No	**69 (8.6)**	738 (91.4)	1.00		1.00	
**Infant death**						
No	**73 (8.3)**	806 (91.7)	1.00		1.00	
**Yes**	**9 (45.0)**	11 (55.0)	**9.04 (3.63–22.52)**	**< .0001**	**7.14 (2.71–18.8)**	**0.0001**
**Maternal CD4+ count** **(cells/mm**^**3**^**)/100**	**82 (9.3)**	803 (90.7)	**0.90 (0.82–0.98)**	**0.02**		
**Log10 of maternal HIV viral load**	**82 (9.2)**	814 (90.8)	**1.88 (1.40–2.53)**	**< .0001**	**1.85 (1.35–2.53)**	**0.0001**

Abbreviations: 3TC = lamivudine; CI, confidence interval; CMV, cytomegalovirus; cCMV = congenital CMV infection CS = Cesarean section; CT = *Chlamydia trachomatis*; HIV, human immunodeficiency virus; NFV = nelfinavir; NG = *Neisseria gonorrhoeae*; NVP = nevirapine; OR, odds ratio; SD, standard deviation; STI = sexually transmitted infection; ZDV = zidovudine.

Detectable CMV from the urine of women and infants were important risk factors for infant HIV acquisition. Women with CMV detected at the time of labor and delivery were over four times more likely to have an HIV-infected infant than those without CMV viruria in adjusted analyses (aOR 4.4, 95% CI 1.5–13.0). Similarly, women whose infants had CMV detected in urine after birth (i.e. congenital CMV; cCMV) were more likely to also have infants with HIV-infected than those without CMV viruria (i.e. without congenital CMV) in the unadjusted analysis (OR 4.1, 95% CI 2.2–7.8), but this did not remain statistically significant in adjusted analysis. (Table 3) Other factors associated with infant HIV acquisition included log_10_ HIV maternal viral load (aOR 1.9, 95% CI 1.4–2.5), illegal substance use during pregnancy (aOR 2.3, 95% CI 1.2–4.6), and subsequent infant death (aOR 7.1, 95% CI 2.7–18.8), whereas higher maternal CD4 count was protective (OR 0.90, 95% CI 0.82–0.98) in the unadjusted analysis. (Table 3)

Further evaluation was done to compare the proportion of maternal STI infections (0 vs 1 vs 2 vs 3 STIs) between HIV-infected and HIV-uninfected infants. (Table 4) Of the 35 HIV-infected infants born to women with STIs (CT, NG, TP, and/or CMV), the majority were those born to women with one STI (n = 24, 68.6%) as opposed to two (n = 10, 28.6%) or three STIs (n = 1, 2.9%). However, significant differences were noted in the proportion of maternal STI infections (0 vs 1, 2 or 3 STIs) and infant HIV-infection, which ranged from 7.5% (47/626) for 0 STIs to 23.8% (10/42) for 2 STIs. Additional regression analyses were done to elucidate these relationships between the number of STIs and HIV MTCT. Women with two STIs were 3.4 times more likely to transmit HIV to their infants than those without STIs (aOR 3.4, 95% CI 1.5–7.7). (Table 3)

**Table 4:** Number of maternal STIs (chlamydia, gonorrhea, syphilis, CMV) Frequency distribution by infant HIV-infection status.

	Infant HIV-infected (N = 82)	Infant HIV-uninfected (N = 817)	p-value* (for total only)
	n (col %)	n (col %)	n (col %)
			**0.003**
**0 STI (n = 626)**	47 (57.3)	579 (70.9)	
**1 STI (n = 223)**			
TP	6 (7.3)	50 (6.1)	
CT	9 (12)	109 (13.3)	
NG		11 (1.4)	
CMV	9 (11)	29 (3.6)	
Total	24 (29.3)	199 (24.4)	
**2 STIs (n = 42)**			
CT, NG	3 (3.7)	13 (1.6)	
CT, TP	2 (2.4)	8 (1.0)	
CT, CMV	5 (6.1)	3 (1.3)	
NG, TP		2 (0.2)	
TP, CMV		6 (0.7)	
Total	10 (12.2)	32 (3.9)	
**3 STIs (n = 8)**			
CT, TP, CMV		1 (0.1)	
CT, NG, CMV	1 (1.2)	3 (0.4)	
CT, NG, TP		3 (0.4)	
Total	1 (1.2)	7 (0.9)	

* *P*-value was generated by the Chi-square test to compare the proportion of STI infections (0 STI vs 1 STI vs 2 STIs vs 3 STIs) between HIV-infected and HIV-uninfected infants.

Abbreviations: CMV, cytomegalovirus and specifically refers to infant congenital CMV here; CT = *Chlamydia trachomatis*; HIV, human immunodeficiency virus; NG = *Neisseria gonorrhoeae*; STI = sexually transmitted infection; TP = *Treponema pallidum* (syphilis).

## Discussion

The results of this study were notable for the high prevalence of overall STIs (TP, CT, NG, and CMV) in this sub-cohort of HIV-infected pregnant women. The presence of at least one of these STIs represented an increased risk for HIV MTCT, particularly two STIs, with CMV being the most important co-infection contributing to HIV perinatal transmission.

In general, limited published studies have focused on evaluating the impact of untreated maternal STIs such as CT, NG, TP, and active CMV infection in pregnancy on HIV mother-to-child transmission (MTCT), nor have they comparatively evaluated the potential contribution of individual co-infections within the same cohort on perinatal HIV transmission. The biological plausibility for STIs increasing HIV MTCT risk was suggested in studies of non-pregnant women which demonstrated increased HIV genital shedding in the presence of STIs.[24, 25] Chorioamnionitis has also been demonstrated to increase the risk of HIV MTCT.[26–33] Selected studies have demonstrated a link between specific STIs with HIV MTCT, including results from our prior individual HPTN 040 analyses.[19–22, 34–37]

Over 30% of HIV-infected pregnant women in the present analysis had at least one STI (TP, CT, NG, and/or CMV). Our findings re-emphasize that women in our study were at high-risk for multiple STIs apart from HIV, particularly younger women. Predictors of STIs such as younger maternal age has been previously well-documented.[38–43] Women enrolled in the parent study were a high-risk group by definition; they were late to present for HIV diagnosis and not on antiretrovirals (ARVs) during pregnancy because they were only diagnosed with HIV at the time of labor and delivery. Nearly 30% did not receive antenatal care and more than a third of women engaged in tobacco, alcohol, and/or illegal substance use during pregnancy.

Collectively, results of this analysis provide additional support that STIs (TP, CT, NG, and/or CMV) increase the risk of HIV MTCT by nearly two-fold and for those with two STIs, possibly more than three-fold. Forty-three percent of infants with HIV were born to women with at least one of these STIs, while rates of HIV MTCT were most pronounced in women with CMV (>26% but possibly as high as >30% for those with CMV viruria) and CT (12.5%) infection.

Our previous studies of individual STIs also demonstrated that these conditions were associated with an increased risk of HIV MTCT. In our earlier individual analyses, 10% of the original NICHD HPTN 040 maternal cohort had positive syphilis results and were twice as likely to transmit HIV to their infants (adjusted OR 2.1, 95% CI 1.3–1.4).[20] Among 1373 HIV-infected pregnant women with high rates of CT (18.1%) and NG (4.6%), CT appeared to pose a possible 1.5-fold increased risk of HIV MTCT (OR 1.5, 95% CI 0.94–2.3).[19] We believe that the present study did not highlight this MTCT risk as clearly due to the reduction in sample size required to perform a combined analysis, which included testing of subjects for all four co-infections. Apart from our own published results from HPTN 040, literature focusing on the impact of STIs (TP, CT, and NG) and the risk of HIV MTCT has also demonstrated mixed results, particularly for CT and NG.[33, 34, 36, 40]

In the present study, maternal CMV (especially CMV viruria) was significantly associated with an increased risk of HIV MTCT (more than four-fold). A prior analysis of the same cohort demonstrated high rates of CMV viruria (9.2%) among HIV-infected pregnant women at the time of delivery, with maternal CMV viruria being associated with a similar but higher risk of HIV MTCT (aOR 5.6, 95% CI 1.9–16.8).[22] The clinical significance of detectable CMV viruria at the time of labor and delivery in HIV-infected pregnant women not on ARVs, however, is not well-understood. Considering that it is very likely that most of the studied women were already CMV seropositive before pregnancy due to the frequent exposure to CMV in these populations, CMV viruria might result from CMV reactivation in the genitourinary tract or reinfection with a new virus strain.[44]

The relationship between HIV and CMV perinatal infections is complex, since infection with one may be a risk factor for infection with the other pathogen. Yet, while several studies have shown that HIV-exposure in pregnancy is a risk factor for congenital CMV, fewer have demonstrated that congenital CMV acquisition is also a risk factor for HIV perinatal transmission.[31, 37, 45, 46] To our knowledge, the only other primary study to address this aspect of the relationship was a retrospective case-control study of 293 HIV-infected and HIV-exposed, uninfected infants from Thailand.[37, 47] They found that congenital CMV was a risk factor for HIV MTCT, particularly for in utero HIV (OR 8.1, 95% CI 1.5–63.7).[37]

In summary, maternal co-infections such as TP, CT, NG, and CMV facilitate HIV perinatal transmission by a variety of means. Genital tract infections from these organisms may lead to cervicitis, which may trigger increased cervico-vaginal HIV viral shedding [24, 25, 48–52] or may cause acute or chronic placental inflammation. [26, 30–34] This inflammation at the maternal-fetal interface may induce immune activation and alter cytokine production, increasing viral load, and upregulating the expression of CCR5 T-cell receptors as well as CCR5 HIV co-receptors on Hofbauer cells (macrophages) in the placenta, which contribute towards increased HIV tropism and infectivity.[46, 53] It has been suggested that many of these factors are especially important in understanding the role CMV infection plays as a risk factor for HIV MTCT, particularly at the placental interface. These pathogens appear to effectively overcome inherent placental antiviral mechanisms that safeguard against fetal infection.[46, 53]

### Study limitations

As discussed previously in our results section, specimen availability for testing for potential co-infections determined inclusion in the present analysis. While study inclusion and exclusion populations were similar, some differences were noted in maternal age, mode of delivery, prenatal care, alcohol use, tobacco use, and maternal HIV viral load. In addition, in the parent study, 28% of subjects came from South Africa and 72% from the Americas. This proportion was not maintained in the sub-analysis as specimen availability was limited for South African subjects. Therefore, 13.8% of subjects were from South Africa and the remainder from the Americas in the present analysis, p < 0.0001. This implies that our results might not be as generalizable to the South African site. Additional limitations include inability to test for other STIs and genital tract infections such as bacterial vaginosis, *Trichomonas vaginalis*, and Herpes simplex virus.

## Conclusion

We conducted this analysis to explore the potential synergy between several co-infections and increased risk for HIV MTCT in a large cohort of HIV-infected mothers and HIV-exposed infants. HIV-infected pregnant women in this cohort were at very high risk for STIs (TP, CT, NG, and/or CMV). The presence of one or more infections nearly doubled the odds of HIV MTCT, and the presence of two co-infections tripled the odds. In particular, CMV in HIV-infected pregnant women, especially the presence of maternal CMV viruria at delivery, appeared to confer the largest risk of perinatal HIV transmission. Our study’s results emphasize the importance that undiagnosed STIs may play in pregnancy as risk factors for HIV MTCT. Our data underscores the need to augment current existing antenatal care programs to include STI screening, particularly for high-risk women such as young, HIV-infected pregnant women as well as ensuring that HIV-infected women have consistent access to antiretroviral treatment and monitoring, particularly during pregnancy.

## Supporting information

S1 Table Comparison of included vs. excluded HPTN 040 study mother-infant pairs. (DOCX)

S2 Table Number of HIV-infected infants (*in utero or intrapartum*) and maternal STI exposure (CT, NG, TP, or CMV). (DOCX)

## Abbreviations

HIV: human immunodeficiency virus
NICHD: National Institute of Child Health and Human Development
HPTN: HIV Prevention Trials Network
STI: sexually transmitted infection
CT: *Chlamydia trachomatis* (chlamydia)
NG: *Neisseria gonorrhoeae* (gonorrhea)
TP: *Treponema pallidum* (syphilis)
CMV: cytomegalovirus
MTCT: mother-to-child transmission
