# Supplementary material for: Combined evaluation of sexually transmitted infections in HIV-infected pregnant women and infant HIV transmission
Source: PLoS One. 2018 Jan 5;13(1):e0189851. doi: 10.1371/journal.pone.0189851 (PMC5755782; doi:10.1371/journal.pone.0189851)
Supplement: S1 Table — (DOCX) [file pone.0189851.s001.docx]

| **Supporting Information**  **S1 Table: Comparison of Included vs. Excluded HPTN 040 Study Mother-Infant Pairs** | | | | |
| --- | --- | --- | --- | --- |
|  | **Total (N=1684)** | **Included (N=899)** | **Excluded (N=785)** | **p-value** |
|  | **n (col %)** | **n (col %)** | **n (col %)** |  |
| **Maternal age** | | | |  |
| Continuous |  |  |  |  |
| **Mean (std. dev.)** | **26.9 (6.3)** | **26.5 (6.3)** | **27.3 (6.3)** | **0.01** |
| Median (min.-max.) | 26 (13-47) | 26 (14-45) | 26 (13-45) |  |
| Categorical |  |  |  |  |
| 13-24 | 667 (39.6) | 376 (41.8) | 291 (37.1) | 0.07 |
| 25-29 | 472 (28.0) | 252 (28.0) | 220 (28.0) |  |
| 30 and older | 545 (32.4) | 271 (30.1) | 274 (34.9) |  |
| **Region** | | | |  |
| **Americas** | **1210 (71.9)** | **775 (86.2)** | **435 (55.4)** | **<.0001** |
| South Africa | 474 (28.1) | 124 (13.8) | 350 (44.6) |  |
| **Mode of delivery** | | | |  |
| **Cesarean before rupture** | **395 (23.5)** | **246 (27.4)** | **149 (19.0)** | **<.0001** |
| Vaginal + CS After rupture | 1288 (76.5) | 653 (72.6) | 635 (81.0) |  |
| **Maternal syphilis (TP)** | | | |  |
| Yes | 153 (9.3) | 78 (8.7) | 75 (10.1) | 0.34 |
| No | 1492 (90.7) | 821 (91.3) | 671 (89.9) |  |
| **Maternal chlamydia (CT) or**  **gonorrhea (NG)** | | | |  |
| No | 1120 (80.1) | 726 (80.8) | 394 (79.0) | 0.42 |
| Yes | 278 (19.9) | 173 (19.2) | 105 (21.0) |  |
| **Maternal chlamydia (CT)** | | | |  |
| No | 1148 (82.1) | 739 (82.2) | 409 (82.0) | 0.91 |
| Yes | 250 (17.9) | 160 (17.8) | 90 (18.0) |  |
| **Maternal gonorrhea (NG)** | | | |  |
| No | 1335 (95.5) | 863 (96.0) | 472 (94.6) | 0.22 |
| Yes | 63 (4.5) | 36 (4.0) | 27 (5.4) |  |
| **Infant CMV (congenital CMV; cCMV)** | | | |  |
| No | 931 (93.6) | 842 (93.7) | 89 (92.7) | 0.72 |
| Yes | 64 (6.4) | 57 (6.3) | 7 (7.3) |  |
| **Infant HIV Status** | | | |  |
| No | 1544 (91.7) | 817 (90.9) | 727 (92.6) | 0.20 |
| Yes | 140 (8.3) | 82 (9.1) | 58 (7.4) |  |
| **Prenatal Care** | | | |  |
| **No** | **631 (37.5)** | **273 (30.4)** | **358 (45.6)** | **<.0001** |
| Unknown | 6 (0.4) | 2 (0.2) | 4 (0.5) |  |
| Yes | 1047 (62.2) | 624 (69.4) | 423 (53.9) |  |
| **Alcohol use during pregnancy** | | | |  |
| **>=1/week** | **282 (16.9)** | **139 (15.6)** | **143 (18.4)** | **0.02** |
| >1/month, <1/wk | 120 (7.2) | 64 (7.2) | 56 (7.2) |  |
| <=1/month | 194 (11.6) | 123 (13.8) | 71 (9.1) |  |
| Never | 1072 (64.3) | 566 (63.5) | 506 (65.2) |  |
| **Tobacco use during pregnancy** | | | |  |
| **>10/day** | **169 (10.1)** | **124 (13.9)** | **45 (5.8)** | **<.0001** |
| 6-10/day | 130 (7.8) | 63 (7.0) | 67 (8.6) |  |
| <=5/day | 240 (14.4) | 144 (16.1) | 96 (12.3) |  |
| Never | 1133 (67.8) | 563 (63.0) | 570 (73.3) |  |
| **Illegal substance use**  **during pregnancy** | | | |  |
| Yes | 149 (8.9) | 88 (9.8) | 61 (7.8) | 0.15 |
| No | 1526 (91.1) | 807 (90.2) | 719 (92.2) |  |
| **Infant death** | | | |  |
| No | 1639 (97.3) | 879 (97.8) | 760 (96.8) | 0.22 |
| Yes | 45 (2.7) | 20 (2.2) | 25 (3.2) |  |
| **Gestation age (weeks)** | | | |  |
| Continuous | 38.6 (1.7) | 38.6 (1.6) | 38.6 (1.7) | 0.40 |
| Mean (std. dev.) | 39 (32-42) | 39 (32-42) | 39 (32-42) |  |
| Median (min.-max.) |  |  |  |  |
| Categorical |  |  |  |  |
| 36 or less | 175 (10.4) | 83 (9.2) | 92 (11.7) | 0.10 |
| 37 or more | 1509 (89.6) | 816 (90.8) | 693 (88.3) |  |
| **Low birth weight (< 2500g)** | | | |  |
| Continuous |  |  |  |  |
| Mean (std. dev.) | 2988.8 (521.8) | 3009.4 (516.7) | 2965.2 (526.8) | 0.06 |
| Median (min.-max.) | 3000 (1510-4850) | 3010 (1595-4410) | 2980 (1510-4850) |  |
| Categorical |  |  |  |  |
| No | 1397 (83.0) | 757 (84.2) | 640 (81.5) | 0.15 |
| Yes | 287 (17.0) | 142 (15.8) | 145 (18.5) |  |
| **Prior history of stillbirth** | | | |  |
| No | 1606 (95.5) | 855 (95.2) | 751 (95.9) | 0.49 |
| Yes | 75 (4.5) | 43 (4.8) | 32 (4.1) |  |
| **Any history of preterm birth** | | | |  |
| No | 1370 (81.7) | 736 (82.4) | 634 (81.0) | 0.44 |
| Yes | 306 (18.3) | 157 (17.6) | 149 (19.0) |  |
| **Any Infant Serious Adverse Event** | | | |  |
| No | 1015 (60.3) | 549 (61.1) | 466 (59.4) | 0.48 |
| Yes | 669 (39.7) | 350 (38.9) | 319 (40.6) |  |
| **Maternal HIV viral load, categorical**  **(copies/mL)** | | | |  |
| Missing | 8 (0.5) | 3 (0.3) | 5 (0.6) | **0.02** |
| <=400 | 110 (6.5) | 52 (5.8) | 58 (7.4) |  |
| 401 to <= 10,000 | 601 (35.7) | 322 (35.8) | 279 (35.5) |  |
| 10,001 to 100,000 | 735 (43.6) | 418 (46.5) | 317 (40.4) |  |
| >100,000 | 230 (13.7) | 104 (11.6) | 126 (16.1) |  |
| **Log10 of maternal HIV viral load** |  |  |  |  |
| Mean (std. dev.) | 4.1 (0.9) | 4.1 (0.8) | 4.0 (1.0) | 0.69 |
| Median (min.-max.) | 4.2 (1.7-6.8) | 4.2 (1.7-6.5) | 4.1 (1.8-6.8) |  |
| **Maternal CD4+ count**  **(cells/mm3)/100** |  |  |  |  |
| Mean (std. dev.) | 514.5 (309.2) | 519.3 (308.8) | 509.0 (309.9) | 0.55 |
| Median (min.-max.) | 459 (12-)2678 | 465 (12-2160) | 448 (17-2678) |  |

Abbreviations: 3TC = lamivudine; CI, confidence interval; CMV, cytomegalovirus; CS=Cesarean section; CT = *Chlamydia trachomatis*; HIV, human immunodeficiency virus; NFV = nelfinavir; NG = *Neisseria gonorrhoeae*; NVP = nevirapine; OR, odds ratio; SD, standard deviation; STI = sexually transmitted infection; ZDV = zidovudine.
