# Supplementary material for: Combined evaluation of sexually transmitted infections in HIV-infected pregnant women and infant HIV transmission
Source: PLoS One. 2018 Jan 5;13(1):e0189851. doi: 10.1371/journal.pone.0189851 (PMC5755782; doi:10.1371/journal.pone.0189851)
Supplement: S2 Table — (DOCX) [file pone.0189851.s002.docx]

**Supporting Information**

**S2 Table: Number of HIV-infected Infants (*In Utero or Intrapartum*)**

**and Maternal STI Exposure (CT, NG, TP, or CMV)**

| **Infant HIV-infection** | **CT** | **NG** | **TP** | **CMV** | **N=35** | **Summary** |
| --- | --- | --- | --- | --- | --- | --- |
| ***In Utero*** | No | No | No | **Yes** | 8 | 8 infants HIV- infected in utero were also infected with CMV but their mothers were CT/NG/TPs negative |
| ***In Utero*** | **Yes** | No | No | **Yes** | 4 | 4 HIV infected in utero infants were also CMV infected and had mothers who were CT positive |
| ***In Utero*** | **Yes** | No | No | No | 5 | 5 HIV infected in utero infants had CT positive mothers |
| ***In Utero*** | **Yes** | No | **Yes** | No | 2 | 2 HIV infected in utero infants had CT and TP positive mothers |
| ***In Utero*** | **Yes** | **Yes** | No | No | 2 | 2 HIV infected in utero infants had CT and NG infected mothers |
| ***In Utero*** | No | No | **Yes** | No | 4 | 4 HIV infected in utero infants had TP positive mothers |
| ***Intrapartum*** | No | No | No | **Yes** | 1 | 1 HIV infected intrapartum infant was also infected CMV |
| ***Intrapartum*** | **Yes** | No | No | **Yes** | 1 | 1 HIV infected intrapartum infant was also infected CMV and had CT positive mother |
| ***Intrapartum*** | **Yes** | **Yes** | No | **Yes** | 1 | 1 HIV infected intrapartum infant was CMV infected and had CT and NG positive mother |
| ***Intrapartum*** | **Yes** | No | No | No | 4 | 4 HIV infected intrapartum infants had positive CT mothers |
| ***Intrapartum*** | **Yes** | **Yes** | No | No | 1 | 1 HIV infected intrapartum infant had CT and NG positive mother |
| ***Intrapartum*** | No | No | **Yes** | No | 2 | 2 HIV infected intrapartum infants had positive TP mother |
|  |  |  |  |  |  |  |

Abbreviations: CMV, cytomegalovirus and specifically refers to infant congenital CMV here. CT = *Chlamydia trachomatis*; HIV, human immunodeficiency virus; NG= *Neisseria gonorrhoeae*; STI = sexually transmitted infection; TP= *Treponema pallidum* (syphilis).
